# Development and validation of the protocol for the evaluation of voice in patients with hearing impairment (PEV-SHI)^☆^

**DOI:** 10.1016/j.bjorl.2019.05.007

**Published:** 2019-07-03

**Authors:** Ana Cristina Coelho, Alcione Ghedini Brasolotto, Fayez Bahmad

**Affiliations:** a Universidade de Brasília, Programa de Pós-Graduação em Ciências da Saúde, Brasília, DF, Brazil; b Universidade de São Paulo, Faculdade de Odontologia de Bauru, Departamento de Fonoaudiologia, Bauru, SP, Brazil

**Keywords:** Voice, Voice quality, Hearing disorders, Hearing loss, Validation studies, Voz, Qualidade da voz, Distúrbios auditivos, Perda de audição, Estudos de validação

## Abstract

**Introduction:**

The voice of individuals with hearing impairment has been widely described, and can be compromised in all levels of the phonatory system.

**Objective:**

To develop and validate an instrument for evaluating the voice of this population.

**Methods:**

The instrument underwent the validation steps suggested by the Scientific Advisory Committee of the Medical Outcomes Trust. The study sample consisted of seventy-eight Brazilian people with cochlear implants (experimental group) and 78 individuals with normal hearing (control group), divided in groups by age range — children from 3 to 5 years; children from 6 to 10 years and adults from 18 to 46 years. The study sample participated in a voice recording of the sustained vowel /a/, connected speech and spontaneous conversation, in which three voice specialists rated using the proposed instrument. It consists of visual-analog scales of suprasegmental aspects, respiratory-phonatory coordination, resonance, phonation, additional parameters and general vocal perception.

**Results:**

Evaluation by an expert committee and a pilot test established content validity. Reliability measures showed excellent test-retest reproducibility for the majority of the parameters. Analysis with the ROC curve showed that perceptual evaluation with the sustained vowel did not strongly differentiate individuals with cochlear implants from those with normal hearing, and the parameter “speech rate” did not differentiate the groups at all. For the connected speech and spontaneous conversation, the majority of the parameters differentiated the experimental group from the control group with an area under the curve ≥0.7. The cutoff values with maximum specificity and sensitivity were 30.5 for mild, 49.0 for moderate and 69.5 for intense deviation.

**Conclusions:**

The protocol for the evaluation of voice in subjects with hearing impairment, PEV-SHI, is a reliable and useful tool for assessing the particularities of the voice of individuals with hearing impairment treated with cochlear implants and can be used in research and clinical settings to standardize evaluation and facilitate information exchange among services.

## Resumo

**Introdução:**

A voz de indivíduos com deficiência auditiva tem sido amplamente descrita e pode estar comprometida em todos os níveis do sistema fonatório.

**Objetivo:**

Desenvolver e validar uma ferramenta para avaliar a voz dessa população.

**Método:**

A ferramenta foi submetida às etapas de validação sugeridas pelo *Scientific Advisory Committee of the Medical Outcomes Trust*. A amostra do estudo foi composta por 78 indivíduos brasileiros com implantes cocleares (grupo experimental) e 78 indivíduos com audição normal (grupo controle) divididos em grupos por faixa etária - crianças de 3 a 5 anos; crianças de 6 a 10 anos e adultos de 18 a 46 anos. Os participantes do estudo realizaram uma gravação de voz da vogal /a/ sustentada, fala encadeada e conversa espontânea, que foi avaliada por três especialistas em voz com o instrumento proposto. Este instrumento consistem em escalas visuais analógicas dos aspectos supras segmentares da voz, coordenação pneumofonoarticulatória, ressoância, fonação, parâmetro adicional e impressão geral da qualidade vocal.

**Resultados:**

A avaliação por um comitê de especialistas e um teste piloto estabeleceram a validade de conteúdo. Medidas de confiabilidade mostraram excelente teste-reteste reprodutibilidade para a maioria dos parâmetros. A análise com a curva ROC mostrou que a avaliação perceptivo-auditiva com a vogal sustentada não diferenciou significantemente os indivíduos com implante coclear daqueles com audição normal e o parâmetro “velocidade de fala” não diferenciou os grupos. Para a fala encadeada e conversa espontânea, a maioria dos parâmetros diferenciou o grupo experimental do grupo controle, com uma área sob a curva ≥ 0,7. Os valores de corte com máxima especificidade e sensibilidade foram 30,5 para desvio discreto, 49,0 para desvio moderado e 69,5 para desvio intenso.

**Conclusões:**

O protocolo de avaliação de voz do deficiente auditivo é uma ferramenta confiável e útil para avaliar as particularidades da voz de indivíduos com deficiência auditiva e implante coclear e pode ser utilizada em pesquisas e contextos clínicos para padronizar a avaliação e facilitar a troca de informações entre os serviços.

## Introduction

Voice production occurs by the integration of the respiratory, phonatory and articulatory systems,^1^, ^2^ also involving highly complex mechanisms related to the central and peripheral nervous systems, such as auditory monitoring.^3^ It can be described through auditory-perceptual, acoustic, aerodynamic evaluations and laryngeal imaging.^3^ The auditory perceptual evaluation is considered the gold standard in voice assessment and enables characterization and quantification of perceptual vocal features.^4^, ^5^

The voice characteristics of individuals with hearing impairment can vary according to the type, severity, onset of the hearing loss, and to the treatment of choice. A list of perceptual attributes used to characterize the voice of these individuals in the last 10 years include: negative overall impression of the voice quality^6^, ^7^, ^8^; roughness^6^; strain^6^, ^9^; resonance disorders^7^, ^10^, ^11^; high pitch^7^; instability^7^, ^12^; and altered suprasegmental features such as intelligibility, articulation^10^ and intonation.^13^ Respiration, phonation, resonance and suprasegmental features are intimately related. For example, many of the references to nasality in deaf speech may refer not only to the actual feature of nasal resonance, but misarticulation of nasals, lack of oral/nasal distinctions, pitch variation, or any combination of these parameters.^14^ These perceived characteristics can be justified by the lack of auditory monitoring of the voice, causing difficulty in developing phonatory control and abilities to regulate and vary the voice use in different situations.^3^, ^15^

Therefore, in addition to social, educational, and language limitations, hearing impairment can cause specific deviation of the communication related to speech and voice, interfering with intelligibility and crucially compromising the social integration of the individual,^3^ so it is important that the assessment of voice production cover all of these elements.

The studies that performed auditory-perceptual evaluation of the voice of individuals with hearing impairment used protocols and scales directed to the global population with voice problems such as the GRBAS scale (G — Grade; R — Roughness; B — Breathiness; A — Asthenia; S — Strain)^16^ and the Consensus Auditory-Perceptual Evaluation of Voice (CAPE-V).^17^ These scales, however, focus on voice production mainly at a glottal level, and therefore do not approach other pertinent features of the voice of the population with hearing impairment, such as the different possible resonance deviations and suprasegmental features of the voice. Also, the lack of standardization of the evaluation process across studies, such as which scale to use and the rating methods, can lead to unreliable and conflicting results.

For an adequate evaluation, it is important that the instrument consider all the relevant parameters to study a specific population. In addition, the scale should allow reliable discrimination between the normal voice and the voice of the target population.^18^ The validation, therefore, of an instrument that approaches the singular voice characteristics of those with hearing impairment can bring important directions for speech-language pathologists regarding the investigation of voice production and rehabilitation of oral communication of these individuals. The purpose of this study was to develop an instrument for evaluating the voice of individuals with hearing impairment who use cochlear implants, establishing its validity for clinical and scientific purposes.

## Methods

The ethics committee of the Brasília University — College of Health Sciences approved this study under process number 16887713.4.0000.0030. All participants, parents or legal guardians signed the informed consent.

### Participants

This study involved the participation of 156 individuals, seventy-eight people with cochlear implants (Experimental Group — EG) and their hearing peers (Control Group — CG) divided in groups by age range: 52 children from 3 to 5 years (G1), 54 children from 6 to 10 years (G2) and 50 adults from 18 to 46 years (G3). Half of the participants of each group consisted of the EG and half consisted of the CG. All participants were native speakers of the Brazilian Portuguese language. The EG included individuals with bilateral, severe to profound sensorineural hearing loss using a cochlear implant, with absence of associated disorders, attending a rehabilitation program, and who had experience of device use of at least one year. This study did not consider other criteria such as hearing loss onset, unilateral or bilateral implant, or use of contralateral hearing aid, since its purpose was to develop an instrument for the overall population with cochlear implants. The CG consisted of individuals with normal hearing. To verify normal hearing, the participants of the CG underwent pure-tone threshold audiometry. Exclusion criteria for both groups were professional voice use, stage of menopause for women, current or previous smoking, regular use of alcoholic drinks, previous laryngeal surgery and being ill with pulmonary or upper airway infection on the day of the recording session.

### Validation steps

The criteria recommended by the Scientific Advisory Committee of the Medical Outcomes Trust^19^ directed the development and validation process of the instrument. The validation steps include describing the conceptual and measurement model, determining reliability measures, content validity, construct validity, interpretability and describing respondent and administrative burden.

### Conceptual and measurement model

The *Protocolo de Avaliação de Voz do Deficiente Auditivo* (PAV-DA), translated as Protocol for the Evaluation of Voice in Subjects with Hearing Impairment (PEV-SHI) (Appendix A) was developed by consensus between three speech-language and hearing sciences professionals, based on perceptual features studied in the literature that stand out in the voice of individuals with hearing impairment. The voice tasks selected were the sustained vowel /a/, connected speech (numbers from 1 to 10) and spontaneous conversation. A 100 mm or 200 mm Visual-Analog Scale (VAS) follows each parameter. For the 100 mm line, the leftmost portion reflected the absence of deviation and the right end of the scale reflected the judgment of most intense deviation. For the parameters intonation, speech rate, pitch and loudness a 200 mm line was used, since the nature of the deviation can turn to opposite sides. For example, the pitch can be either too low or too high. Therefore, in the 200 mm scale, the midpoint was defined as adequate, with possible deviations to the left or right of this midpoint, allowing the rater to visualize the full range of the deviation in the VAS. Suprasegmental features and respiratory-phonatory coordination were to be assessed only for the spontaneous conversation. The selected parameters and their respective definitions were:

• Suprasegmental aspects of the voice quality:
  ∘ Intelligibility: How understandable the speech is;
  ∘ Articulation: The correct production of speech sounds;
  ∘ Intonation: The melodic pattern and frequency variation in speech;
  ∘ Speech rate: How fast or slow speech in produced within a sentence.
• Respiratory-phonatory coordination: Coordination between breath and speech.
• Resonance: The way in which the voice is projected into space. It may have an isolated or mixed characteristic. The raters selected more than one item in the protocol in case of a mixed resonance. The term “excessively” was used to express unbalance and predominance of the resonance in a certain region of the vocal tract. The resonance was classified as:
  ∘ Excessively laryngeal: Low resonance focus, the voice seems to be stuck in the throat;
  ∘ Excessively pharyngeal: The resonance focus is not so low. It is more centered in the oropharynx, which gives the voice a metal feature;
  ∘ Excessively hyponasal: Insufficient use of nasal cavity, which causes a perception of nasal obstruction. This parameter must be disregarded in the evaluation of the sustained vowel /a/;
  ∘ Excessively hypernasal: Excessive use of the nasal cavity, which causes a perceived nasal voice;
  ∘ Excessively anterior: Oral resonance focus, which causes a perception of a childlike voice in adults. In case of children, their voices do not match their ages. It seems like the person places their tongue anteriorly during speech;
  ∘ Excessively posterior: The resonance focus is in the posterior oral space, resembling someone speaking with a hot potato in the mouth.
• Phonation:
  ∘ Strain: Excessive phonatory effort;
  ∘ Breathiness: Audible air escape in the voice;
  ∘ Roughness: Irregularity in voicing source;
  ∘ Instability: Unstable quality of emission regarding frequency and/or intensity. The same emission can have short-term or long-term instability. Both should be considered;
  ∘ Pitch: Perceptual correlate of fundamental frequency. A medium pitch is neither too low nor too high, and varies based on gender and age. The deviation may occur to high or low;
  ∘ Loudness: Perceptual correlate of intensity. A medium loudness is neither too loud nor too soft, considering the environmental features. The deviation may occur to loud or soft;
• Additional parameter: Any other relevant vocal characteristic the rater may notice and which is not addressed in the protocol.
• General vocal perception: Global, integrated perception of voice deviation, after every parameter is separately assessed. The general vocal perception involves all aspects assessed in the protocol.

### Content validity

The establishment of content validity consisted of two steps. In the first, an expert committee consisting of speech-language pathologists and audiologists, who were not involved in in the development of the protocol, judged the initial version of the PEV-SHI for its clarity, parameters and form of evaluation. All suggestions were analyzed and a partial version was determined. In the second, two speech-language pathologists with 20 years of training performed a pilot test based on the analysis of five voice samples of each speech task of individuals with cochlear implants using the instrument. Both had participated in the expert committee. After the pilot test, final adjustments were made, determining the final version of the PEV-SHI.

### Data collection and auditory perceptual evaluation

After the determination of the final version of the PEV-SHI, three voice specialists who had not participated in any of the previous steps of this study rated the voice samples with it. The use of an odd number of raters is important to avoid potential ties in the evaluation and this number of raters was selected based on common practice in auditory-perceptual assessment of voice.^20^, ^21^, ^22^, ^23^, ^24^, ^25^

The three raters had extensive experience in performing perceptual evaluation in normal and disordered voices, and one of them had experience working with voice disorders in individuals with cochlear implants. The raters participated in previous training sessions, with the purpose of becoming acquainted with the protocol and having the same understanding of the parameters assessed in each speech task. The ratings were performed separately by age range and speech tasks. The raters knew the age and gender of each voice sample, but not if it belonged to a participant of the EG or the CG. The raters were also unfamiliar with the patients. Each rater performed the task individually and the data was charted. If the difference between the score given by the three raters for a determined parameter was within a margin of ten points, the mean of the three scores was considered. For the parameters which the difference exceeded 10 points, consensus rating was carried out. The raters gathered in additional meetings for new analysis, discussion and rating of these parameters.

The voice samples were recorded with the Sony Sound Forge 10.0 software with sampling rate of 44.100 Hz, 16 Bit, and Mono channel. The head microphone AKG C512, pre-amplifier M-audio Fast Track Pro and a notebook were used. The procedure was performed in a quiet, soundproof room with the microphone positioned at 45° with a 3 cm distance from the participant’s mouth.

### Reliability

In order to establish reliability of the PEV-SHI, the raters repeated the auditory-perceptual evaluation of 20% of the voice samples in random order. The Interclass Correlation Coefficient (ICC) was used to verify test-retest reproducibility. The following correlation scale was adopted:

### Construct validity

Construct validity was determined in two steps. First, by comparing the scores of the EG and CG using ANOVA. In addition, analysis of efficiency, sensibility and specificity were performed using the ROC curve. The closer the AUC is to 1.0, the greater the distinction between the EG and the CG. Second, by correlating the scores of the PEV-SHI with an external clinical criterion. For this, the raters, in another occasion classified the voice samples according to the overall dysphonia Grade (G) of the GRBAS scale. This score was compared to the score of the general vocal perception of the PEV-SHI.

### Interpretability

The cutoff values were determined based on the score of the overall dysphonia Grade (G) and on the levels of specificity and sensitivity given by the ROC curve to differentiate the voice of an individual with hearing impairment from a listener using the score of the general vocal perception of the PEV-SHI for the three voice samples. In addition to the cutoff values, the severity of the vocal deviation was determined, which can range from a normal variability of the voice quality, mild deviation, moderate deviation or intense deviation.

### Burden

Respondent and administrative burden included a full description of any demands involving the administration of the PEV-SHI, including time, training and necessary resources.

## Results

This study presents the development and validation process of the Protocol for the Evaluation of Voice in Subjects with Hearing Impairment (PEV-SHI).

### Content validity

After analysis of all the suggestions made by the expert committee and the pilot tests, the final version of the PEV-SHI was determined. Changes from the initial version included changes in definition and order of presentation of parameters, change in terminology and unification of the parameters articulation and instability, which were previously unraveled into more parameters. The final version is clear, comprehensible and contains adequate content for the target population.

### Reliability

Table 1 illustrates the results of the ICC for the three groups together, showing excellent reliability for all tasks and excellent test-retest reproducibility. For the groups separately, only one parameter presented with poor correlation. For G1 in the sustained vowel and for G3 in the connected speech there was poor correlation for the parameter strain. Correlation was either good, or in most cases, excellent, for all parameters in all tasks in the separate groups.

**Table 1:** Interclass Correlation Coefficient – ICC by parameter/task for all groups together.

All groups	Sustained vowel		Connected speech		Spontaneous conversation	
	ICC	*p*-Value	ICC	*p*-Value	ICC	*p*-Value
Intelligibility	‒	‒	‒	‒	99.7%	<0.001^a^
Articulation	‒	‒	‒	‒	99.4%	<0.001^a^
Intonation	‒	‒	‒	‒	99.0%	<0.001^a^
Speech rate	‒	‒	‒	‒	94.8%	<0.001^a^
Coordination	‒	‒	‒	‒	97.9%	<0.001^a^
Laryngeal	94.5%	<0.001^a^	90.2%	<0.001^a^	98.2%	<0.001^a^
Pharyngeal	97.2%	<0.001^a^	89.9%	<0.001^a^	96.1%	<0.001^a^
Hyponasal	‒	‒	91.1%	<0.001^a^	95.4%	<0.001^a^
Hypernasal	89.8%	0.001^a^	95.0%	<0.001^a^	96.1%	<0.001^a^
Anterior	86.7%	0.003^a^	79.6%	<0.001^a^	88.2%	<0.001^a^
Posterior	96.8%	<0.001^a^	88.3%	<0.001^a^	97.6%	<0.001^a^
Strain	76.6%	0.021^a^	93.3%	<0.001^a^	97.2%	<0.001^a^
Breathiness	85.4%	0.004^a^	94.0%	<0.001^a^	64.5%	0.003^a^
Roughness	82.4%	0.008^a^	86.5%	<0.001^a^	94.6%	<0.001^a^
Instability	87.7%	0.002^a^	91.4%	<0.001^a^	98.0%	<0.001^a^
Pitch	98.5%	<0.001^a^	94.0%	<0.001^a^	88.2%	<0.001^a^
Loudness	97.9%	<0.001^a^	94.1%	<0.001^a^	94.9%	<0.001^a^
General perception	86.7%	0.003^a^	98.1%	<0.001^a^	99.2%	<0.001^a^

a *p* < 0.05.

### Construct validity

The comparison of the scores between the EG and CG using ANOVA showed significant differences in most parameters. The task with least significant results was the sustained vowel, followed by the connected speech and spontaneous conversation (Table 2, Table 3).

**Table 2:** Comparison of the Protocol for the Evaluation of Voice in Subjects with Hearing Impairment — PEV-SHI parameters between EG1 and CG1, and between EG2 and CG2 for all emissions.

		Group 1						Group 2					
		Sustained vowel		Connected speech		Spontaneous conversation		Sustained vowel		Connected speech		Spontaneous conversation	
		Mean	*p*-Value	Mean	*p-*Value	Mean	*p*-Value	Mean	*p*-Value	Mean	*p*-Value^a^	Mean	*p*-Value
Inteligibility	EG	‒	‒	‒	‒	69.4	<0.001^a^	‒	‒	‒	‒	80.7	<0.001
	CG	‒		‒		0.6		‒		‒		2.7	
Articulation	EG	‒	‒	‒	‒	68	<0.001^a^	‒	‒	‒	‒	77	<0.001
	CG	‒		‒		10.6		‒		‒		16.2	
Intonation	EG	‒	‒	‒	‒	0.6	0.926	‒	‒	‒	‒	44.3	<0.001
	CG	‒		‒		1.2		‒		‒		0.2	
Speech rate	EG	‒	‒	‒	‒	−9.9	0.061	‒	‒	‒	‒	−2.5	0.416
	CG	‒		‒		−0.3		‒		‒		0.6	
Coordination	EG	‒	‒	‒	‒	49.7	<0.001^a^	‒	‒	‒	‒	46.6	<0.001
	CG	‒		‒		24.2		‒		‒		27.4	
Laryngeal	EG	37.3	0.05^a^	49.2	<0.001^a^	51.7	<0.001^a^	43.4	0.028^a^	50.2	<0.001	40.5	<0.001
	CG	33.7		30.9		27.5		35.3		20.3		24.8	
Pharyngeal	EG	27.3	<0.001^a^	37.6	<0.001^a^	51.4	<0.001^a^	42.6	0.088	54.9	<0.001	46.9	<0.001
	CG	18		22.3		22.3		34.9		19.9		25.2	
Hyponasal	EG	‒	‒	34	<0.001^a^	33.3	<0.001^a^	‒	‒	26.6	<0.001	30.4	<0.001
	CG	‒		4.8		6.4		‒		4.2		15.4	
Hypernasal	EG	26.1	0.005^a^	41.8	<0.001^a^	55.9	<0.001^a^	45.2	0.15	51.4	<0.001	48.9	<0.001
	CG	15.7		19.8		20.3		37.8		19.3		25.4	
Anterior	EG	12.2	0.074	28.1	<0.001^a^	38.1	<0.001^a^	12.9	0.915	24.7	<0.001	16.3	0.017
	CG	7.6		10.6		7.7		13.4		9.2		6.3	
Posterior	EG	22.7	0.017^a^	38.6	<0.001^a^	25.3	<0.001^a^	24.2	0.031^a^	28	<0.001	18.9	<0.001
	CG	13.5		2.9		0.4		10.8		0		1.4	
Strain	EG	35.3	0.005^a^	48.6	<0.001^a^	53.8	<0.001^a^	44.3	0.119	56.2	<0.001	50.4	<0.001^a^
	CG	30.1		25.9		23.3		36.7		20.2		26	
Breathiness	EG	24.3	0.005^a^	27	0.115	35.4	<0.001^a^	34.9	0.366	31.9	<0.001	32.1	<0.001^a^
	CG	33.5		23.2		22		32.2		20.2		22.1	
Roughness	EG	23.4	0.675	30.2	0.002^a^	33.3	<0.001^a^	37.5	0.015^a^	32	<0.001	36.1	<0.001^a^
	CG	24.4		22.6		21.1		28.9		19.9		22.6	
Instability	EG	37.5	0.001^a^	46.6	<0.001^a^	51.1	<0.001^a^	44.8	0.005^a^	46	<0.001	53.6	<0.001^a^
	CG	28		21.7		16		31.1		18.1		20.2	
Pitch	EG	11.8	0.05^a^	17.6	0.003^a^	28.5	<0.001^a^	5.2	0.787	14.8	0.015	10.3	0.038^a^
	CG	4.6		3		2.2		3.9		3.4		2.4	
Loudness	EG	0.1	0.007^a^	1.7	0.091	6	0.075	12.8	0.16	12.6	0.001	4.1	0.062
	CG	−9		−4.7		1.2		5.2		−1.5		−3.4	
General perception	EG	38.8	0.235	52.7	<0.001^a^	69.8	<0.001^a^	48.5	0.083	60.8	<0.001	77.9	<0.001^a^
	CG	36.3		27.6		25		41		24.1		27.1	

EG, Experimental Group; CG, Control Group.

a *p* <  0.05.

**Table 3:** Comparison of the protocol for the evaluation of voice in subjects with hearing impairment ‒ PEV-SHI parameters between EG3 and CG3, and between EG and CG for all emissions.

		Group 3						All					
		Sustained vowel		Connected speech		Spontaneous conversation		Sustained vowel		Connected speech		Spontaneous conversation	
		Mean	*p*-Value	Mean	*p*-Value	Mean	*p*-Value	Mean	*p*-Value	Mean	*p*-Value	Mean	*p*-Value
Inteligibility	EG	‒	‒	‒	‒	15.9	0.001^a^	‒	‒	‒	‒	55.7	<0.001^a^
	CG	‒		‒		0		‒		‒		1.1	
Articulation	EG	‒	‒	‒	‒	33.2	<0.001^a^	‒	‒	‒	‒	59.6	<0.001^a^
	CG	‒		‒		0		‒		‒		9.1	
Intonation	EG	‒	‒	‒	‒	9.4	0.08	‒	‒	‒	‒	17.6	<0.001^a^
	CG	‒		‒		−0.6		‒		‒		0.3	
Speech rate	EG	‒	‒	‒	‒	−3.4	0.271	‒	‒	‒	‒	−5.4	0.021^a^
	CG	‒		‒		0.9		‒		‒		0.4	
Coordination	EG	‒	‒	‒	‒	33	<0.001^a^	‒	‒	‒	‒	43.3	<0.001^a^
	CG	‒		‒		11.2		‒		‒		21.1	
Laryngeal	EG	35.4	0.33	33.4	0.009^a^	35.2	<0.001^a^	38.6	0.005^a^	44.4	<0.001^a^	42.7	<0.001^a^
	CG	32.4		22.8		22.4		33.9		24.8		25	
Pharyngeal	EG	26	0.015^a^	28.2	<0.001^a^	23.8	0.011^a^	31.9	<0.001^a^	40.2	<0.001^a^	41	<0.001^a^
	CG	17.3		12.1		13.4		23.4		18.3		20.4	
Hyponasal	EG	‒	‒	25.8	<0.001^a^	27	<0.001^a^	‒	‒	28.9	<0.001^a^	30.3	<0.001^a^
	CG	‒		2.4		0.5		‒		3.8		7.5	
Hypernasal	EG	23.6	0.233	32.4	<0.001^a^	41.2	<0.001^a^	31.5	0.011^a^	41.9	<0.001^a^	48.9	<0.001^a^
	CG	17.3		4.8		13.6		23.6		14.8		19.9	
Anterior	EG	6	0.945	7.5	0.848	7.7	0.366	10.4	0.54	20.3	<0.001^a^	21.2	<0.001^a^
	CG	6.2		6.8		4.4		9.1		8.9		6.2	
Posterior	EG	18.7	0.014^a^	30	<0.001^a^	37	<0.001^a^	21.9	<0.001^a^	32.4	<0.001^a^	27	<0.001^a^
	CG	7.1		4.1		4.6		10.5		2.3		2.1	
Strain	EG	40.9	0.002^a^	37.3	<0.001^a^	41.3	<0.001^a^	40	<0.001^a^	47.4	<0.001^a^	48.7	<0.001^a^
	CG	31.4		18.1		19.8		32.8		21.5		23.1	
Breathiness	EG	24.4	0.198	8.3	0.298	17	0.04^a^	27.8	0.045^a^	22.5	0.004^a^	28.3	<0.001^a^
	CG	28.9		5.5		10.2		31.6		16.5		18.2	
Roughness	EG	37.9	0.051	21.2	0.108	31.8	0.003^a^	32.7	0.021^a^	27.8	<0.001^a^	33.7	<0.001^a^
	CG	31.5		16.2		19.8		28.2		19.6		21.2	
Instability	EG	46.2	0.003^a^	25.2	0.001^a^	43.2	<0.001^a^	42.7	<0.001^a^	39.5	<0.001^a^	49.3	<0.001^a^
	CG	35.6		10		18.1		31.5		16.8		18.1	
Pitch	EG	9	0.157	4.1	0.452	7.9	0.302	8.7	0.038^a^	12.3	0.001^a^	15.9	<0.001^a^
	CG	0.8		−0.8		1.8		3.2		1.9		2.2	
Loudness	EG	7.3	0.002^a^	0.9	0.299	8.8	0.007^a^	6.5	<0.001^a^	5	<0.001^a^	6.3	<0.001^a^
	CG	−10.6		−2.2		−2.5		−4.8		−2.8		−1.5	
General perception	EG	45.1	0.004^a^	47.2	<0.001^a^	52.9	<0.001^a^	44	0.001^a^	53.5	<0.001^a^	66.9	<0.001^a^
	CG	36.5		21.5		20.1		37.9		24.5		24.1	

EG, Experimental Group; CG, Control Group.

a *p* < 0.05.

The efficiency of the PEV-SHI, given by the Area Under the Curve (AUC) of the ROC curve, demonstrated that the majority of the parameters is adequate to differentiate individuals with hearing impairment from individuals with normal hearing, especially for the connected speech and spontaneous conversation. Table 4 presents the AUC for each parameter for the separated groups and for the groups together. Table 5 illustrates the cutoff values and highest levels of sensitivity and specificity for each parameter for all of the groups together.

**Table 4:** Area Under the Curve (AUC) by parameter/task for Group 1 (G1), Group 2 (G2), Group 3 (G3) and all groups together.

	Sustained vowel				Connected speech				Spontaneous conversation			
	G1	G2	G3	All	G1	G2	G3	All	G1	G2	G3	All
Intelligibility	‒	‒	‒	‒	‒	‒	‒	‒	0.962	0.973	0.7	0.873
Articulation	‒	‒	‒	‒	‒	‒	‒	‒	0.996	0.962	0.94	0.939
Intonation	‒	‒	‒	‒	‒	‒	‒	‒	0.503^a^	0.913	0.567^a^	0.659^a^
Speech rate	‒	‒	‒	‒	‒	‒	‒	‒	0.369^b^	0.418^b^	0.39^b^	0.396^b^
Coordination	‒	‒	‒	‒	‒	‒	‒	‒	0.945	0.92	0.83	0.878
Laryngeal	0.672^a^	0.678^a^	0.592^a^	0.639^a^	0.931	0.954	0.693^a^	0.846	0.959	0.913	0.892	0.899
Pharyngeal	0.826	0.642^a^	0.692^a^	0.685^a^	0.859	0.945	0.798	0.856	0.978	0.936	0.712	0.842
Hyponasal	‒	‒	‒	‒	0.914	0.827	0.802	0.845	0.86	0.836	0.792	0.823
Hypernasal	0.713	0.63^a^	0.557^a^	0.613^a^	0.868	0.923	0.81	0.859	0.966	0.927	0.918	0.93
Anterior	0.628^a^	0.49^b^	0.445^b^	0.51^a^	0.805	0.749	0.53^a^	0.673^a^	0.893	0.639^a^	0.533^a^	0.682^a^
Posterior	0.682^a^	0.66^a^	0.66^a^	0.668^a^	0.89	0.86	0.77	0.842	0.789	0.738	0.881	0.8
Strain	0.724	0.634^a^	0.748	0.691^a^	0.924	0.975	0.919	0.927	0.975	0.942	0.93	0.945
Breathiness	0.301^b^	0.589^a^	0.386^b^	0.412^b^	0.643^a^	0.792	0.607^a^	0.625^a^	0.914	0.799	0.665^a^	0.761
Roughness	0.468^b^	0.703	0.659^a^	0.593^a^	0.744	0.912	0.6^a^	0.739	0.918	0.916	0.731	0.848
Instability	0.757	0.713	0.742	0.726	0.914	0.942	0.774	0.84	0.996	0.992	0.914	0.97
Pitch	0.615^a^	0.526^a^	0.62^a^	0.591^a^	0.704	0.652^a^	0.574^a^	0.644^a^	0.798	0.598^a^	0.579^a^	0.661^a^
Loudness	0.715	0.636^a^	0.737	0.682^a^	0.608^a^	0.713	0.57^a^	0.626^a^	0.593^a^	0.624^a^	0.684^a^	0.635^a^
General perception	0.586^a^	0.629^a^	0.738	0.65^a^	0.949	0.953	0.914	0.934	0.986	0.978	1	0.989

a AUC > 0.5 < 0.7.

b AUC ≤ 0.5.

**Table 5:** Cutoff values, sensitivity and specificity by parameter/task for all groups together.

All	Sustained vowel			Connected speech			Spontaneous conversation		
	CV	SE	SP	CV	SE	SP	CV	SE	SP
Intelligibility	‒	‒	‒	‒	‒	‒	19.5	74.0%	100%
Articulation	‒	‒	‒	‒	‒	‒	29.5	80.5%	98.7%
Intonation	‒	‒	‒	‒	‒	‒	15	58.4%	89.7%
Speech rate	‒	‒	‒	‒	‒	‒	15	18.2%	93.6%
Coordination	‒	‒	‒	‒	‒	‒	32.5	80.5%	90.0%
Laryngeal	35.5	61.0%	64.1%	35.5	70.1%	92.3%	31.5	75.3%	94.9%
Pharyngeal	20.5	81.8%	53.8%	30.5	70.1%	91.0%	34.5	70.1%	97.4%
Hyponasal	‒	‒	‒	16.5	72.7%	91.0%	18.5	76.6%	83.3%
Hypernasal	26.5	58.4%	66.7%	27.5	71.4%	97.4%	31	88.3%	94.9%
Anterior	33.5	9.1%	97.4%	27	44.2%	94.9%	28.5	42.9%	98.7%
Posterior	19.5	55.8%	76.9%	17.5	71.4%	96.2%	22	61.0%	97.4%
Strain	32.5	74.0%	61.5%	29.5	85.7%	88.5%	34.5	87.0%	98.7%
Breathiness	60.5	1.3%	100%	23.5	51.9%	70.5%	30.5	51.9%	94.9%
Roughness	26.5	66.2%	52.6%	27.5	55.8%	87.2%	25.5	76.6%	82.1%
Instability	32.5	75.3%	60.3%	29.5	68.8%	92.3%	28.5	92.2%	96.2%
Pitch	16.5	39.0%	88.5%	17.5	44.2%	87.2%	24	41.6%	94.9%
Loudness	7.5	37.7%	88.5%	11.5	31.2%	97.4%	16.5	26.0%	98.7%
General perception	44.5	49.4%	78.2%	39.5	80.5%	97.4%	33.5	97.4%	93.6%

CV, cutoff value; SE, sensitivity; SP, specificity.

Regarding the correlation between scores of the PEV-SHI with an external clinical criterion, there are significant and positive correlation between the scores of the G parameter of the GRBAS scale and the score of the general vocal perception of the PEV-SHI, indicating that as the general vocal perception increases in the VAS, it increases in the numerical scale (NS) and vice-versa. Most correlations were classified as good and excellent. This analysis was not performed for the connected speech and spontaneous conversation for G3, since there was no variability of responses in the NS (Table 6).

**Table 6:** Correlation between the parameter “general vocal perception” of the Protocol for the Evaluation of Voice in Subjects with Hearing Impairment ‒ PEV-SHI with the parameter Grade (G) of the GRBAS scale.

Group (G)		Sustained vowel		Connected speech		Spontaneous conversation	
		Corr (r)	*p*-Value	Corr (r)	*p*-Value	Corr (r)	*p*-Value
G1	Experimental	83.7%	<0.001^a^	82.6%	<0.001^a^	94.3%	<0.001^a^
	Control	74.1%	<0.001^a^	79.3%	<0.001^a^	71.9%	<0.001^a^
G2	Experimental	95.7%	<0.001^a^	93.8%	<0.001^a^	94.5%	<0.001^a^
	Control	91.5%	<0.001^a^	82.4%	<0.001^a^	81.6%	<0.001^a^
G3	Experimental	77.5%	<0.001^a^	62.6%	0.001^a^	87.3%	<0.001^a^
	Control	34.2%	0.094	- x -	- x -	- x -	- x -
All	Experimental	84.5%	<0.001^a^	80%	<0.001^a^	94%	<0.001^a^
	Control	67.8%	<0.001^a^	73%	<0.001^a^	63.9%	<0.001^a^

Corr, correlation.

a *p* < 0.05.

### Interpretability

To determine interpretability of the PEV-SHI the ROC curve was used to set the cutoff values based on the parameter general perception of the voice quality and the parameter G from the GRBAS scale. The cutoff values in the VAS were obtained by correlation between the VAS and the NS (Table 7). The maximum efficacy rule was used to estimate the cutoff values, considering the highest values of sensitivity and specificity, which were concomitantly combined with the highest values of efficiency. The cutoff values were obtained by group and task. In some cases, the analysis was not performed because there was no variability of responses in the NS (Table 7).

**Table 7:** AUC, cutoff values, sensitivity and specificity for the parameter general voice deviation of the Protocol for the Evaluation of Voice in Subjects with Hearing Impairment — PEV-SHI.

Group (G)	Speech task	Deviation	AUC	CV	SE	SP
G1	Sustained vowel	Mild	0.936	34.5	81.8%	100%
		Moderate	0.997	43	100%	94.4%
		Intense	- x -	- x -	- x -	- x -
	Connected speech	Mild	1	28.0	100%	100%
		Moderate	0.995	42	95.5%	100%
		Intense	1	75	100%	100%
	Spontaneous conversation	Mild	1	36	100%	100%
		Moderate	0.98	53.5	96%	100%
		Intense	1	71	100%	100%
G2	Sustained vowel	Mild	0.978	35.5	95.2%	100%
		Moderate	1	52.5	100%	100%
		Intense	0.968	69	100%	91.3%
	Connected speech	Mild	1	37	95.7%	100%
		Moderate	1	54	100%	100%
		Intense	0.982	72	100%	94.4%
	Spontaneous conversation	Mild	0.976	30.5	100%	100%
		Moderate	1	50	100%	100%
		Intense	0.957	78	89.5%	100%
G3	Sustained vowel	Mild	0.957	37.5	52.6%	100%
		Moderate	0.843	- x -	- x -	- x -
		Intense	1	- x -	- x -	- x -
	Connected speech	Mild	- x -	26.5	95.7%	100%
		Moderate	1	49.5	80%	100%
		Intense	0.989	70.5	100%	100%
	Spontaneous conversation	Mild	0.728	- x -	- x -	- x -
		Moderate	- x -	47.0	100%	100%
		Intense	- x -	69.5	100%	95.7%
All	Sustained vowel	Mild	0.893	37.5	67.7%	100%
		Moderate	0.939	43.5	100%	81%
		Intense	0.987	69	100%	97.3%
	Connected speech	Mild	0.978	31.5	94.4%	100%
		Moderate	0.942	49.5	85.5%	100%
		Intense	0.987	72.5	100%	97%
	Spontaneous conversation	Mild	1	30.5	100%	100%
		Moderate	0.998	49.9	96.7%	100%
		Intense	0.997	69.5	100%	95.5%

AUC, Area Under the Curve; CV, cutoff value; SE, sensitivity; SP, specificity.

### Burden

Respondent burden refers to the recording procedure of the voice samples. In this study, the PEV-SHI was used to assess the voice of 156 individuals, divided into the EG and CG by age range. The individual had to attend the location of the recording where they received instructions to perform the three tasks. The time for the recording was about 10 min. The PEV-SHI was considered not suitable for individuals with hearing impairment who had poor language development and could not perform the speech tasks. Administrative burden included a quiet environment, recording equipment (computer, sound card and microphone), headphones, a printed PEV-SHI, pencil and ruler. The time for analysis by the raters was about two minutes for each speech task. To complete the PEV-SHI the rater must be familiar with all of the definitions and instructions for completing the analysis with the protocol. The rater must also be experienced in the evaluation of normal and altered voices, and with speech and voice of people with cochlear implants.

## Discussion

Auditory-perceptual evaluation of the voice quality is a key element in the clinical assessment of the voice. The use of non-specific instruments, however, may not approach some relevant characteristics of a certain population. The population with hearing impairment is an example of people with particular voice features the exceed alterations at a glottal level. An instrument that approaches all of the potential attributes of the voice is, therefore, of great importance to characterize with precision the voice of this population.

The validation process was conducted in steps.^19^ Based on the process of development, revision and pilot test, content validity was established. By the definition of content validity,^26^, ^27^ the PEV-SHI addresses in a relevant and representative way the voices of subjects with hearing impairment with cochlear implants and is adequate for its elements of instructions, parameters and scoring.

In the following steps, auditory-perceptual evaluation was performed with the PEV-SHI for the extraction of the psychometric measures of reliability, efficiency, sensitivity, specificity and cutoff values.

Reliability measures, extracted with the ICC based of the repetition of the auditory-perceptual evaluation of 20% of the sample showed good and excellent reliability for the majority of the parameters (Table 1). The PEV-SHI has good test-retest reproducibility, and therefore, is considered a reliable instrument.^19^

The comparison of the EG with the CG using ANOVA (Table 2, Table 3) evidenced significant differences for most parameters. The task with less significant differences was the sustained vowel. With the variance analysis alone, it is not possible to determine whether these results were due to the voice characteristics of the populations or to the sensitivity of the PEV-SHI, since this test compares means between the populations.^28^ Measures given by the ROC curve (Table 4) complemented and corroborated this analysis. The ROC curve represents the relationship between the sensitivity and the specificity of any given test.^29^ The AUC measures the performance (efficiency) of the test, in this case, its accuracy to identify individuals with hearing impairment. The closer the AUC is to 1.0, the better the ability of the instrument to perform an adequate classification as to what it proposes to evaluate. A test that is not able to discriminate between individuals with or without a certain disorder has an AUC of 0.5.^29^ In this study, parameters with AUC ≤ 0.5 were considered not suitable for distinguishing IC users and listeners. Values ​​between 0.5 and 0.7 were considered acceptable and values ≥0.7 were considered adequate. There were cases of AUC ≤ 0.5 for isolated parameters of the PEV-SHI in all of the groups.

In the sustained vowel, there was occurrence of AUC ≤ 0.5 for the parameters breathiness (G1), anterior resonance (G2 and G3) and breathiness (G3 and all) (Table 4). The sustained vowel is a test of glottal efficiency,^30^ essentially evaluating the ability of an individual to control the aerodynamic forces of the pulmonary airflow and myoelastic forces of the larynx^31^ and does not suffer interference of suprasegmental features of the voice. Stability is an important featured to be evaluated and, in fact, this was the only parameter of the PEV-SHI that had AUC ≥ 0.7 for all groups in this task (Table 4). The parameters that least differentiated the CG from the EG were breathiness and anterior resonance. Most of the remaining parameters presented with AUC > 0.5 and <0.7 for the sustained vowel.

The PEV-SHI was more efficient to differentiate the population with CI from the population with normal hearing for the tasks involving speech. Even though the parameters pitch and loudness had acceptable AUC in the connected speech and spontaneous conversation for most groups (Table 4), these parameters have great clinical relevance, are easily interpreted and are routinely used in voice assessment.^17^ Breathiness is an expected feature in children and women due to laryngeal configuration.^32^ The same occur with roughness in the male voice.^33^ Although these parameters do not strongly distinguish the EG from the CG, they are important for the PEV-SHI since they are expected voice characteristics for determined age and gender, regardless of the hearing loss.

The same occurs with the parameters resonance and intonation. Resonance had AUC ≥ 0.7 for two groups in the connected speech and one group in the spontaneous conversation (Table 4). Individuals with hearing loss tend to present resonance disorders, since the lack of auditory monitoring leads them to use inadequate vocal tract adjustments in the voice production. A mixed resonance is a common feature,^3^ and for this reason, the PEV-SHI sought to approach the all possible types of resonance. Intonation disorders is a perceived feature of the voice if individuals with hearing loss,^34^, ^35^ however this parameter differentiated the EG from the CG with AUC > 0.5 < 0.7 for three groups and AUC ≥ 0.7 for one group. For every group the AUC for the parameter speech rate was ≥0.5, so it was excluded from the protocol.

The sensitivity and specificity of an instrument refer to its ability to correctly detect individuals, respectively, with or without a disorder.^36^ The results presented on Table 5 suggest that the PEV-SHI is susceptible to error, especially in the sustained vowel. These errors occur when a normal hearing individual is classified as an individual with hearing impairment (false positive) and vice versa (false negative).

To determine construct validity, a simple scale for auditory-perceptual evaluation with power of discrimination of different degrees of vocal deviation using a robust parameter (G of the GRBAS scale) and a unidimensional scale (general vocal deviation of the PEV-SHI) were used.^5^, ^33^ This also allowed correspondence between the VAS and NS (numerical scale)^5^, ^20^, ^33^, ^37^^,^^38^ and understanding the boundaries between normal and disordered voices between the EG and CG. Findings showed significant and positive correlation between the scores of the G parameter of the GRBAS scale and the score of the general vocal perception of the PEV-SHI (Table 6).

**Figure 1:**
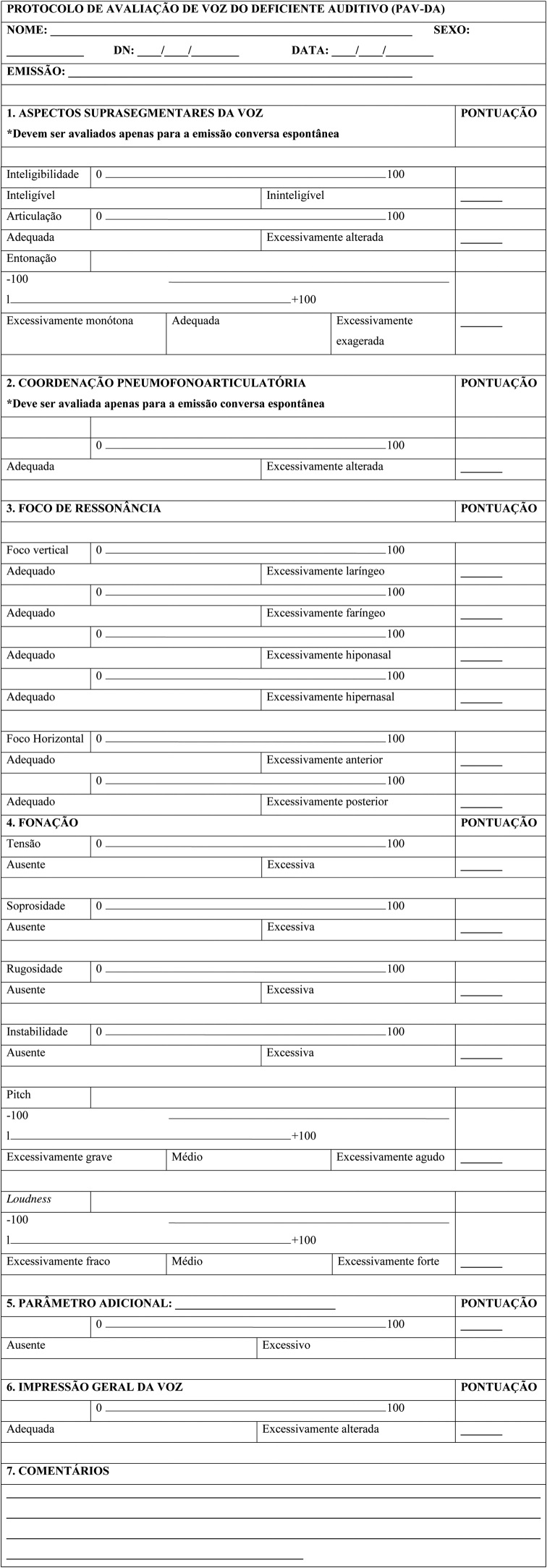
Cutoff values of the PEV-SHI in the VAS (VN, normal variability; MI, mild; MO, moderate; IN, intense).

The cutoff value is a number from which the result of a test is classified either as positive (presence of deviation, disorder or illness that is being tested) or negative (absence of what is being tested). If the result found is smaller than the cutoff value, the result of a test is classified as negative and vice versa.^39^ Depending on the group and task, the PEV-SHI presented with different cutoff values to differentiate the CG from the EG, with AUC close to 1.0 and satisfactory values of sensitivity and specificity (Table 7). This discriminatory power can assure reliable use of these measures on clinical and scientific contexts.^29^ As the results of Table 7 show, cutoff values vary with the speech task, the parameter^5^, ^33^ and age range. In practice, however, it is suggested that the rater use the most robust cutoff values to distinguish the voice of individuals with hearing impairment, providing greater reliability in the use of this instrument for the population with cochlear implants. These results were obtained for all of the groups together in the spontaneous conversation (Table 7). For the PEV-SHI, therefore, the 30.5 value corresponds to the cutoff point between normal variability and mild vocal deviation; the 49.0 value corresponds to the cutoff point between mild and moderate vocal deviation; and the 69.5 value corresponds to the cutoff point between moderate and intense deviation (Fig. 1).

The results discussed in this section show that the sustained vowel did not differentiate the voices of the individuals with cochlear implants from those with normal hearing as robustly as the connected speech and spontaneous conversation. Even so, the vowel can be used, with caution, for evaluation with the PEV-SHI, considering that this task has great importance for the global comprehension of the vocal behavior.^30^, ^40^

Some of the benefits of using the PEV-SHI for the target population over existing auditory-perceptual tools include: evaluating voice while taking into account in a single instrument all elements of the voice production (respiration, phonation, resonance and suprasegmental aspects)^1^, ^2^, ^3^; the possibility to unravel the resonance and evaluate predominance of one or more resonance focus; assessing instability; having a VAS for an additional parameter and assessing the general vocal perception after taking into account all of the parameters.

Although this validation study was performed with CI users, the PEV-SHI can also be of great contribution other groups of individuals with hearing impairment, such as users of hearing aids or other implantable devices. The extraction of psychometric measures for other groups with hearing loss is recommended, since the cutoff values established in this study correspond to CI users of the studied age rage. Further studies include also the use of the PEV-SHI with individuals with hearing impairment during the stages of puberty and aging. The PEV-SHI is currently undergoing transcultural adaptation for the English language.

The PEV-SHI is a reliable and useful tool for assessing the particularities of the voice of individuals with hearing impairment with cochlear implants and can be used in research to standardize evaluation and facilitate information exchange among services. It can also be used as part of the clinical assessment of patients, which should encompass all aspects of oral communication, from auditory abilities, to language development, orofacial functions and voice production. Finally, it can be useful in defining therapeutic goals, and follow up of the patient.

## Conclusion

The content of the Protocol for the Evaluation of Voice in Subjects with Hearing Impairment (PEV-SHI) is adequate for the intended target population. It has good test-retest reproducibility and is sensible and reliable for all the studied age groups, especially for the connected speech and conversational speech. The cutoff values with maximum sensibility and specificity were those found for the overall population in the conversational speech and these can be used as values of reference in the application of the PEV-SHI. The cutoff values to be considered are, therefore, from 0 to 30.5 normal variability of the voice quality, from 30.6 to 49 mild deviation, from 50 to 69.5 moderate deviation and above 69.5 intense deviation. The use of the PEV-SHI requires adequate sound capture, clinical experience and familiarity of the rater with the voice of individuals with hearing impairment.

## Conflicts of interest

The authors declare no conflicts of interest.

## Figures and Tables

**Table:** 

**Protocolo de avaliação de voz do deficiente auditivo (PAV-DA)**
**Apresentação e instruções**
Este protocolo é uma ferramenta para a avaliação perceptivo-auditiva da voz de deficientes auditivos. Seu principal objetivo é descrever a severidade de atributos perceptivo-auditivos da voz dessa população. Deverá será preenchido a partir de três amostras de fala:
Vogal sustentada /a/ com duração de 3 a 5 segundos;
Fala encadeada – contagem de números de 1 até 10 e;
Fala espontânea com duração média de 30 segundos. Sugere-se o tema “me conte sobre um dia especial para você” para adolescentes e adultos. Pra crianças sugere-se dramatizações de histórias infantis conhecidas. Caso não seja possível devido o nível de desenvolvimento da linguagem, o avaliador fica livre para coletar a amostra de fala que for possível.
Os parâmetros para a avaliação são:
**Aspectos suprasegmentares da voz**
a) Inteligibilidade: O quanto a fala é compreensível.
b) Articulação: Produção correta dos sons da fala.
c) Entonação: Padrão de melodia e variação da frequência na fala.
**Coordenação pneumofonoarticulatória:** Coordenação entre respiração e fala.
**Foco de ressonância:** A forma como a voz é projetada no espaço. Pode ter característica isolada ou mista. No caso de ressonância mista, o avaliador pode marcar mais de um item no protocolo. A ressonância pode ser classificada como:
a) Excessivamente laríngea: foco de ressonância baixo, a voz parece estar presa na garganta.
b) Excessivamente faríngea: foco não tão baixo, mais centrado na orofaringe, dando uma característica metálica à voz.
c) Excessivamente hiponasal: uso insuficiente da cavidade nasal, dando sensação de obstrução nasal.
c) Excessivamente hipernasal: uso excessivo da cavidade nasal, dando sensação de voz fanhosa.
d) Excessivamente anterior: foco ressonantal oral, dando uma sensação de voz infantilizada em adultos, e uma voz que não corresponde a idade em crianças, dando a impressão de língua anteriorizada.
e) Excessivamente posterior: som posteriorizado, dando uma sensação de batata quente na boca.
**Fonação**
a) Tensão: Esforço fonatório excessivo.
b) Soprosidade: Escape de ar audível na voz.
c) Rugosidade: Irregularidade na fonte sonora.
d) Instabilidade: Qualidade instável da emissão em termos de frequência e/ou intensidade. A instabilidade pode ser de curto ou longo prazo dentro de uma mesma emissão, sendo que ambas devem ser consideradas.
e) *Pitch*: Correlação perceptiva da frequência fundamental. Entende-se por *pitch* médio aquele que não evidencia característica nem muito grave nem muito agudo para determinado sexo e idade. O desvio pode ser para o grave ou para o agudo.
f) *Loudness:* Correlação perceptiva da intensidade. Entende-se por *loudness* média aquela que não evidencia característica nem muito forte nem muito fraca para determinado sexo e idade, levando em consideração as características do ambiente. O desvio pode ser para o forte ou para o fraco.
**Parâmetro adicional**: Qualquer outra característica vocal relevante que o avaliador perceba e que não é abordado no instrumento.
**Impressão geral da voz:** Impressão global, integrada do desvio da voz, após avaliação de todos os parâmetros separadamente. A impressão geral da voz envolve todos os aspectos abordados no instrumento: aspectos suprasegmentares, coordenação pneumofonoarticulatória, foco de ressonância, fonação e parâmetro adicional (se houver).
Cada um desses parâmetros é acompanhado de uma escala visual analógica de 100 mm ou de 200 mm, dependendo de sua natureza. Para a escala de 100 mm o avaliador deverá indicar o grau de desvio da normalidade utilizando uma pequena marcação (risco), considerando a linha como crescente, ou seja, quando mais à direita, maior o desvio. Na escala de 200 mm, o ponto médio foi definido como médio ou adequado, com desvios possíveis para direita ou para esquerda deste ponto intermediário. A pontuação será medida com uma régua em mm e deverá ser anotada à direita de cada parâmetro. Comentários ou parâmetros adicionais podem ser acrescentados no fim do protocolo. Uma ficha de avaliação deverá ser preenchida para cada tarefa fonatória.

**Table:** 
