# Erratum on “Development and validation of the protocol for the evaluation of voice in patients with hearing impairment (PEV-SHI)”

**DOI:** 10.1016/j.bjorl.2021.06.001

**Published:** 2021-07-15

**Authors:** Ana Cristina Coelho, Alcione Ghedini Brasolotto, Fayez Bahmad

**Affiliations:** a Universidade de Brasília, Programa de Pós-Graduação em Ciências da Saúde, Brasília, DF, Brazil; b Universidade de São Paulo, Faculdade de Odontologia de Bauru, Departamento de Fonoaudiologia, Bauru, SP, Brazil

In the article “**Development and validation of the protocol for the evaluation of voice in patients with hearing impairment (PEV-SHI)**”, published in Braz J Otorhinolaryngol. 2020;86:748-62, with DOI number https://doi.org/10.1016/j.bjorl.2019.05.007, in the title of the article, where it reads:

**Development and validation of the protocol for the evaluation of voice in patients with hearing impairment (PEV-SHI)**

It should read:

**Development and validation of the protocol for the evaluation of voice in subjects with hearing impairment (PEV-SHI)**

